# Patients with pathogenic variants for breast cancer other than BRCA1 and BRCA2: qualitative interviews about health care experiences

**DOI:** 10.1186/s13053-019-0132-6

**Published:** 2019-12-16

**Authors:** Kristin E. Clift, Sarah K. Macklin, Stephanie L. Hines

**Affiliations:** 1 Center for Individualized Medicine, Mayo Clinic, 4500 San Pablo Rd, Jacksonville, FL 32224 USA; 2 Department of Clinical Genomics, Mayo Clinic, 4500 San Pablo Rd, Jacksonville, FL 32224 USA; 3 Internal Medicine, Mayo Clinic, 4500 San Pablo Rd, Jacksonville, FL 32224 USA

**Keywords:** Familial cancer, Genetic counseling, Genetic risk, Genetics, Hereditary breast cancer, Qualitative research, *PALB2*, *PTEN*, *ATM*, *TP53*, *NBM, RAD51C*, *MSH6*

## Abstract

**Background:**

Genetic testing for hereditary cancer syndromes has been revolutionized by next-generation sequencing, which allows for simultaneous review of numerous genes. Multigene panels are regularly offered to patients because of their scope and decreased cost and turnaround time. However, many genes included on larger panels have not been studied as extensively as *BRCA1* and *BRCA2* (*BRCA1/2*), and their clinical effects are often not as well established.

**Methods:**

We identified patients who received positive test results for pathogenic variants of breast cancer genes from January 2012 through May 2018. We mailed a survey and conducted qualitative interviews to explore the personal and health care experiences of patients with pathogenic variants of *BRCA1/2* and patients with “other” (ie, non-*BRCA1/2* or *PALB2; PTEN; ATM; TP53; NBM, RAD51C; MSH6*) variants. We compared the experiences of these patients.

**Results:**

Fifty-nine out of 128 individuals responded to the survey (46%). Thirty-two patients had *BRCA1/2* variants, and 27 had other variants. (49 women and 10 men; median [range] age, 63 [34–87] years). We interviewed 21 patients (17 women and 4 men; median [range] age, 59.6 [34–82] years). Of the interview participants, ten patients had *BRCA1/2* variants, and 11 had non-*BRCA1/2* variants. Patients reported receiving poor information about their genetic test results, and they often educated their physicians about their results. Some patients believed that they had been ignored or “brushed off” by health care professionals because non-*BRCA1/2* genes are less understood outside the genetics research community. Patients with *BRCA1/2* variants had similar problems with health care providers, despite increased awareness and established guidelines about *BRCA1/2*.

**Conclusions:**

Research is required to understand the clinical significance and proper management of diseases attributable to newly characterized hereditary cancer genes. Additional evaluation of patient and provider education should be at the forefront of efforts to improve patient care.

## Background

Since the 1990s, genetic testing has been offered to patients at high risk for pathogenic variants of the *BRCA1* and *BRCA2* (*BRCA1/2)* genes [1, 2]. Strong evidence shows how pathogenic variants of these two genes affect health, and thorough, well-researched management guidelines exist [3, 4]. Furthermore, widely reported celebrity cases have increased knowledge about genetic tests for *BRCA1/2* in the general population [5, 6].

However, pathogenic changes in *BRCA1/2* do not account for all cases of hereditary breast cancer, and additional genes are associated with an increased cancer risk [7–9]. Many newly discovered genes are now included on panels for hereditary cancer gene testing. These larger panels have become widely available because of the development of next-generation sequencing, a technology that allows for simultaneous assessment of multiple genes. In addition, the advent of direct-to-consumer genetic testing options has increased the likelihood that patients may undergo genetic testing without direct involvement of their physicians. The exact cancer risks of many newly discovered genes are not always known, and some genes appear to convey a moderately increased risk of breast cancer compared with the high risk associated with *BRCA1/2* [4, 10, 11]. Although the association of these other genetic variants with a characteristic of increased breast cancer risk may be apparent (even if that level of risk has not been quantified), often it is unclear which other organs may have an increased risk of malignancy [4, 12]. The ambiguity can impair decision-making about whether or when to initiate interventions, such as increased surveillance, prophylactic surgery, pharmacologic risk reduction, or a combination of these therapies [13]. Furthermore, physicians do not have clear guidance to help counsel patients about the risks or management of pathogenic variants of other genes [14]. Overall, physician knowledge about genetics is insufficient, and most do not feel comfortable counseling patients about genetic test results [15–17]. Despite growth in the consumer genetic testing industry, general public knowledge about genetics is also limited [18].

The purpose of this study was to assess gaps in physician knowledge and physician discomfort in managing these risks as perceived by patients who had received a diagnosis of a pathogenic variant of a newly characterized gene. We conducted qualitative interviews with patients who had pathogenic variants of *BRCA1/2* genes and patients with pathogenic variants of various “other” (ie, non-*BRCA1/2*) genes to explore their personal and health care experiences, identify gaps in care, and understand differences and similarities in the experiences between these 2 groups of patients.

## Methods

We developed a survey and interview guides which were approved by the Mayo Clinic Institutional Review Board. We identified adult patients (age ≥ 18 years) who had genetic testing services and received reports suggesting hereditary cancer risk. These patients had been referred by their health care professionals (institutional or local providers) to the Department of Clinical Genomics at Mayo Clinic (Jacksonville, Florida) for assessment. We reviewed client utilization reports and identified all patients who received a positive test result for a *BRCA1/2* only test, hereditary breast cancer panel, and hereditary breast and gynecologic cancer gene panel from January 2012 through May 2018; genes included varied based on laboratory and date ordered. The Mayo Clinic Survey Research Center mailed surveys to these patients. The cover letter included a consent form and the invitation to participate in the interview portion of the study. If the patient indicated interest, he or she was contacted by study personnel to schedule an interview. Interested patients completed a private telephone or in-person interview at Mayo Clinic at their convenience. Patients were reminded of the purpose of the study and the consent form. Patients were compensated $25 for their participation in the interview. Interviews were conducted from March 2018 through July 2018.

We conducted semi-structured interviews, which were audio recorded, transcribed, and assigned a unique identification number. An inductive and integrated approach was used to analyze the transcripts. Two coders identified major themes, separately coded the transcripts, and came to consensus through discussion [19–21]. After considering the coded materials, we identified the major themes of the participants’ responses.

Survey topics included demographic information, self-report of genetic testing results and cancer diagnoses, additional measures taken as a result of genetic testing, attitudes about planning for the future (planning for own health, getting support, and having children), and negative feelings about test results. For the purpose of this manuscript, survey results were tested for association with the following condition: having a pathogenic variant in the *BRCA1* or *BRCA2* gene.

Frequency and percentage of respondents are presented for all variables except age, for which median and range are shown. Associations with the condition described above were tested using Fisher’s Exact test for categorical variables, Wilcoxon Rank Sum test for ordinal variables, and Kruskal-Wallis test for continuous variables. *P*-values of < 0.05 were considered to be statistically significant and all tests were two-sided. Statistical analyses were conducted using SAS version 9.4 (SAS Institute, Cary, North Carolina, USA).

## Results

The Mayo Clinic Survey Research Center mailed surveys to 128 patients. Fifty-nine individuals (46%) responded to the survey. Forty-nine respondents were female, and 10 were male. Median age was 63 with range between 34 and 87. Forty-nine of the respondents identified solely as Caucasian, and the remainder identified as African American (6), Asian (2), Native American (1) or Hispanic (1). Thirty two (54%) had a pathogenic variant in *BRCA1/2.* Three of these 32 individuals had another pathogenic variant as well; these second variants were in *CHEK2, NBN* and *MUTYH*. Another individual had both a pathogenic *PALB2* and *CHEK2* variant. The remaining individuals all had one pathogenic variant in the following genes, *CHEK2* (8), *ATM* (7), *PALB2* (5), *TP53* (2), *NBN* (1), *MSH6* (1), *PTEN* (1), and *RAD51C* (1). Eleven of the 59 (19%) reported no history of cancer before or since completing genetic testing. Breast cancer was the most common cancer diagnosis, present in 33 individuals (56%). This was followed by ovarian (7), lung (3), colorectal (3), melanoma (2), uterine (3), prostate (1), renal (1), pancreas (1), thyroid (1), squamous cell carcinoma (1) and serous carcinoma of unknown origin (1). Nine patients had more than one cancer diagnosis.

Patients who had pathogenic variants in *BRCA1/2* were more likely to have received prophylactic surgery (15/29, 52%) than those with a variant in another gene (5/25, 20%) (*p* = .024). There were no statistically significant differences between the two groups for questions that asked about management, planning for the future, increased anxiety, lack of information, or regret.

Of the survey responders who were interested in participation of the interview portion, we interviewed 21 patients (17 women and 4 men; median [range] age, 59.6 [34–82] years). Ten patients had a pathogenic *BRCA1/2* variant, and 11 had a pathogenic variant of a gene other than *BRCA1/2*. (See Table 1) Four interviews took place in person, and the others were completed via telephone.

Patients with pathogenic variants in genes other than *BRCA1/2* were often frustrated by their encounters with local health care providers. We grouped their experiences into several themes.

**Table 1:** Interview Patient Demographic Information (*N* = 21)

Gene	Women/Men, No.	Age, Mean (Range), y
*BRCA1/2*	8/2	51 (34–72)
*PALB2*	4/0	67.8 (55–76)
*ATM*	1/2	63 (48–82)
*CHEK2*	2/0	55 (54–56)
*TP53*	1/0	35
*PTEN*	1/0	38

### Complex cases

Several patients expressed the feeling that their genetic test results made their health care management too complicated for their local physician. This feeling is shown by the following quotes:

I used to go to [a local physician]. He’s a sweet man, a good doctor … but I felt like my body was getting a little more complicated than everyday checkups yearly.

72-year-old woman with *PALB2*-positive breast cancer

I do have a primary care physician in [town], and my oncologist is local, too … the doctors [here] are okay, but … if you baffle them, they don’t offer any suggestions. And, you know, I guess I baffle them a lot.

54- year-old woman with *CHEK2*-positive breast cancer

A patient, who was concerned about passing on pathogenic variants for cancer to his children, described his experience seeking information from a local health care provider:

I called a guy here, my family physician or my internist, and he said there’s nothing around here. They don’t fool with that around here.

81-year-old man with *ATM*-positive pancreatic cancer.

Another patient described her physicians’ reactions to finding out her genetic test results:

I think it’s a new thing for everybody, the *PALB2*. I would say two of my doctors did not know about *PALB2*. They say, “It’s new to me, I didn’t know about that one.” … They pretty much said, “OK, get in touch with the oncologist,” but nothing else.

55- year-old woman with *PALB2*-positive breast cancer

### Brushed off by physicians

Patients often felt that their health care providers dismissed them or the genetic test results they presented, as described by one patient:

I took my report to my oncologist … who I had not been with I think for about 3 years … he did have a geneticist in his office, which was different from when I used to see him. And they kind of pooh-poohed it. I felt like I was discounted—he didn’t want to see me. He usually was a doctor that was very thorough around asking history, but the geneticist just took the paper work and put it somewhere. Same way with my internal medicine doctor.

76-year-old woman with *PABL2*-positive breast cancer

Another patient described her experience when she told her health care providers about her genetic test results:

They didn’t really do much with it. They were unaware … I’ve just learned that, unfortunately, you have to educate yourself because a lot of the medical community just doesn’t really know much about it, and they just, in my experience, have just pushed me aside.

59-year-old woman with *ATM*-positive breast cancer

Another patient described how she was met with little more than a platitude and no additional advice:

Yes, I shared them at the clinic with the physician that I generally see. I did give him that information, absolutely. I gave him a copy of my information. … Basically, there is no particular advice. By the way, I also shared it with my gynecologist. … He didn’t say a whole lot. He just told me that it was unfortunate.

68-year-old woman with *PALB2*-positive breast cancer

### Additional research by physicians

Not all physicians were dismissive. Some patients explained how their physicians learned more about their genetic variants to help them:

[My doctor] got all my information … after I gave it to her, I don’t know how much she had heard about it before, but I know that she then went and researched a bunch, and we met after and talked a lot about it.

35-year-old woman with *TP53*-positive breast cancer

My primary care physician, yes, I took them [the genetic test results] back. But what I’ve found is that most doctors are not familiarized with the *PTEN* mutation, and they’re like, “OK, well, let’s research this a little.”

38-year-old woman with *PTEN*-positive endometrial cancer

### Educating others

Patients with positive test results for variants of genes other than *BRCA1/2* often educated other patients and helped guide their care. One patient described her motivation to assert her knowledge and to help guide the care of other patients:

I feel like I’ve been trying to educate people in this particular facility, as well as patients, about the awareness of genetic testing and the importance … I think that it’s important to just educate the medical community that, hey, this is what’s going on, and … there might be more targeted medications or treatments that you could try instead of what you might be used to. So, I guess for myself, I’ve taken on the role of “Why did I have cancer?” Well, I have cancer because I’m going to help educate people about what’s out there. I’m a teacher by training, so I think I’ll never lose that.

59-year-old woman with *ATM*-positive breast cancer

Another patient noted how people she spoke with did not know about other genes associated with breast cancer:

A lot of people are just, like, clueless about *CHEK2*, and [I] even had to spell it for people, “C-H-E-K-2, the number 2”. … They just say, “Wow, that’s interesting because I hadn’t heard anything about that particular gene, because there’s so much out there about *BRCA1* and *BRCA2*. Even with that actress who had that particular gene and went ahead and did all that double mastectomy.”

56-year-old woman with *CHEK2*-positive breast cancer

### *Patients With* BRCA1/2 *Variants Had Similar Problems*

Even patients with *BRCA1/2* variants reported that their local providers were not particularly interested in their genetic test results or in ordering genetic tests.

Well, I’m looking for a new outside provider because I really didn’t like the doctor I was going to. I brought all of my testing … and he kind of just handed them back to me and said, “OK, well, I don’t need this.” So, I’m looking for another doctor. I just didn’t like the fact that he handed those tests back to me saying, “I really don’t need these.” So, I am looking for another doctor who will be interested in my problems.

72-year-old woman with the *BRCA2* variant

One patient without a personal history of cancer asked about genetic testing services because of her family history. Her physician did not think her genetic risk was a concern:

I had my previous gynecologist, and she didn’t really think it was any type of concern. She brushed it off. And I think when she brushed it off, I didn’t think very much of it.”

62-year-old woman with the *BRCA2* variant

Another patient had undergone *BRCA1/2* testing 5 years prior, but the test results were negative. Her sister mentioned updates in genetic testing, but she was disappointed that she had to mention additional testing instead of her health care providers suggesting it:

When I asked, they were, you know, “Sure,” and insurance covered it. But I think if I didn’t ask, I wouldn’t know, and I was disappointed that it hadn’t been brought to me, instead of me asking because my sister mentioned something.”

62-year-old woman with *BRCA1*-positive breast cancer

When she received a positive result for a pathogenic variation of *BRCA1*, she decided to undergo prophylactic surgery.

Another patient explained that when she asked about genetic testing in the 1990s, her physicians did not think genetic testing was necessary because it was not routine. Now, she believes that physicians should recommend genetic testing to everyone with breast cancer:

I had asked my oncologist up north about it because they were just finding out about this *BRCA* gene. And they said, “Well, I don’t think that’s necessary.” I mean, they were very good physicians, don’t get me wrong, I’m not criticizing them, but I think they felt back in the 90s they know less than they know now. … I just feel that physicians, when they diagnose someone with breast cancer, should probably recommend genetic testing. A lot of them don’t.

70-year-old woman with *BRCA2*-positive breast cancer

## Discussion

Patients with a genetic predisposition to cancer encounter several challenges in their efforts to incorporate this knowledge into their medical care. Consistent observations from the current study include a lack of provider knowledge about the discovery of new cancer genes, associated risks, and clinical options for management. Patients also expressed an awareness of their physicians’ discomfort in managing these risks and an appreciation for those physicians who made additional efforts to learn.

Interestingly, those with pathogenic variants of newly characterized non- *BRCA* genes experienced many of the same barriers as those with pathogenic variants of *BRCA1/2*, despite increased public awareness and medical knowledge about these more established genes.

Such findings suggest an increasing gap between the pace of medical advances in genetics and the ability of providers to incorporate this information into clinical practice. Changes in health care have expanded the understanding of the genetic basis of disease, the advent of next-generation sequencing has enabled commercial laboratories to expand the availability of their clinical offerings, and a single test can now provide results for multiple known cancer-causing genes. In addition, direct-to-consumer tests are available that bypass the health care provider entirely. As a consequence, the results of these tests can be difficult to interpret by both patients and health care providers alike because genetic testing technology outpaces the rate of medical research needed to understand associated risks and to develop evidence-based clinical treatment strategies.

Within the general medical community, knowledge about genetic risk factors for breast cancer remains limited [17]. Although more information exists about the risks and management options associated with *BRCA1/2*, many regularly tested genes have received less attention from the media and the research community. General public awareness is low, and more importantly, patients and their health care providers may be uncertain about how these results affect cancer risk or ongoing care [22]. This lack of certainty is exemplified by the observations of the patients in this study who were frustrated when they attempted to proactively manage their own genetic risk.

Care guidelines for patients with pathogenic *BRCA1/2* variants are well established [4] as confirmed by the survey findings that more BRCA1/ patients received prophylactic surgery. However, gaps remain in physician understanding about management recommendations, and these gaps may be more acute for less well-known genes. Pathogenic variants in these newly described genes are often less common, which can affect the ability of researchers to accurately predict overall lifetime risks and to define optimal clinical strategies for screening and prevention. This uncertainty, in turn, results in a lack of clinical evidence that physicians can use in the everyday care of their patients. Our qualitative research confirms these issues.

This study has several limitations. The number of patients interviewed was small, and the study cohort was recruited from only one tertiary care medical center. Additional research is needed to confirm whether these results occur in other patient populations and to the medical community at large.

However, the data presented do suggest that, when confronted with unfamiliar information, health care providers should consider taking proactive steps, such as reaching out to colleagues or consulting the literature; however, consulting the literature may not always be feasible given the amount of time available for most physicians to devote to each patient.

Physicians should receive education about how to refer a patient to a specialist who can provide management recommendations and how to access informative resources. Greater knowledge about care or management recommendations for health care professionals may help to improve patient experience. The National Comprehensive Cancer Network has practice guidelines for multiple genes related to the risk of hereditary breast cancer [4]. These guidelines are free, outline management recommendations to consider, and emphasize when available research is too limited to recommend changes in care. In an effort to remain pertinent, these guidelines are updated regularly, often at least once per year. Health care providers who are unable to refer patients to a high-risk breast cancer clinic can review these guidelines to determine whether their patients should receive individualized care.

## Data Availability

The datasets used and/or analyzed during the current study are available from the corresponding author on reasonable request.

## Abbreviations

*BRCA1/2*: *BRCA1* and *BRCA2*
